# Construction of IgG–Fab^2^ bispecific antibody via intein-mediated protein trans-splicing reaction

**DOI:** 10.1038/s41598-023-43110-0

**Published:** 2023-09-25

**Authors:** Risa Yamada, Ishin Nakahara, Izumi Kumagai, Ryutaro Asano, Takeshi Nakanishi, Koki Makabe

**Affiliations:** 1 Graduate School of Science and Engineering, Yamagata University, 4-3-16 Jyonan, Yonezawa, Yamagata 992-8510 Japan; 2 Department of Biotechnology and Life Science, Graduate School of Engineering, Tokyo University of Agriculture and Technology, 2-24-16 Naka-cho, Koganei, Tokyo 183-8538 Japan; 3 Division of Science and Engineering for Materials, Chemistry and Biology, Department of Chemistry and Bioengineering, Graduate School of Engineering, Osaka Metropolitan University, Sugimoto 3-3-138, Sumiyoshi-ku, Osaka, 558-8585 Japan

**Keywords:** Proteins, Biochemistry, Medical research, Molecular medicine

## Abstract

A bispecific antibody (bsAb) is a class of engineered antibody molecules that simultaneously binds to two different antigens by having two kinds of antigen-binding domains. One of the major obstacles for the bsAb production is the incorrect chain-pairing problem, wherein each heavy and light chain should form pairings with the correct counterpart’s chains, but the structural similarity of the incorrect partners also forms the incorrect pairings. This study aimed to demonstrate a bsAb construction method using intein-mediated protein *trans*-splicing to create IgG–Fab^2^–type bsAbs, which is a modified antibody with a structure in which two additional Fabs are linked to the N-terminus of the heavy chain of an IgG molecule. The chain-paring problem between a heavy chain and a light chain is circumvented by separate expression and purification of the IgG part and the Fab part. We found that the deletion of a possible glycosylation residue improved the reaction yield and side-reaction cleavage in the protein ligation step. The resulting bsAb, IgG–Fab^2^ (Her2/CD3), demonstrated target binding activity and cytotoxicity mediated by activated T cells. These results indicate that the use of the protein ligation to produce the IgG–Fab^2^ type bsAb will expand the bsAb production method.

## Introduction

A bispecific antibody (bsAb) is an engineered antibody having two different antigen-binding portions within one molecule, while general monoclonal antibodies (mAbs) target only one target antigen^1–4^. The dual binding ability of bsAbs has multiple applications, which cannot be achieved by general mAbs, including recruiting killer immune cells to cancer cells^2^ and activation of receptor molecules by co-cauterization^5^. Such ability makes bsAbs an emerging class of new antibody therapeutics. One difficulty for immunoglobulin G (IgG) bsAb development is a chain-pairing problem that four different polypeptide chains, consisting of two heavy chains and two light chains, should form correct pairings with each other, where only one combination out of 10 combinations is the correct pairing, although it has great potential. Several antibody engineering techniques have been developed to overcome this chain-pairing problem, such as knobs-into-holes mutation for heavy chain pairing, which introduces convex–concave mutations on the interface of the Fc dimer^6^ and CrossMab for heavy chain-light chain pairing, achieved by exchanging the order of domains in the Fab region^7^.

A split intein-mediated protein ligation can be used for generating bsAb molecules among such antibody engineering methods. The reaction, termed protein trans-splicing (PTS), is a widely used protein engineering technique to connect separately expressed two target proteins^8–10^. In the PTS reaction, the N-terminal and the C-terminal part of a split intein (intein-N and intein-C) are fused to the target proteins and ligated with each other to form a peptide bond, and the intein moiety is released without any structural trace at the ligation site. Connecting two single-domain nanobodies is the simplest usage of the ligation technique for the bsAb construction. We previously reported the construction of tandem VHHs in a bacterial cell^11^. Various combinations of tandem VHH bsAb can be created using this method. We further utilized the ligation technique to construct circularly connected VHH bsAb by ligating the N- and C-terminus^12^. The intein-mediated ligation between one Fab arm and the rest of the IgG molecule was also reported for constructing IgG-type bispecific antibodies^13–15^.

**Figure 1:**
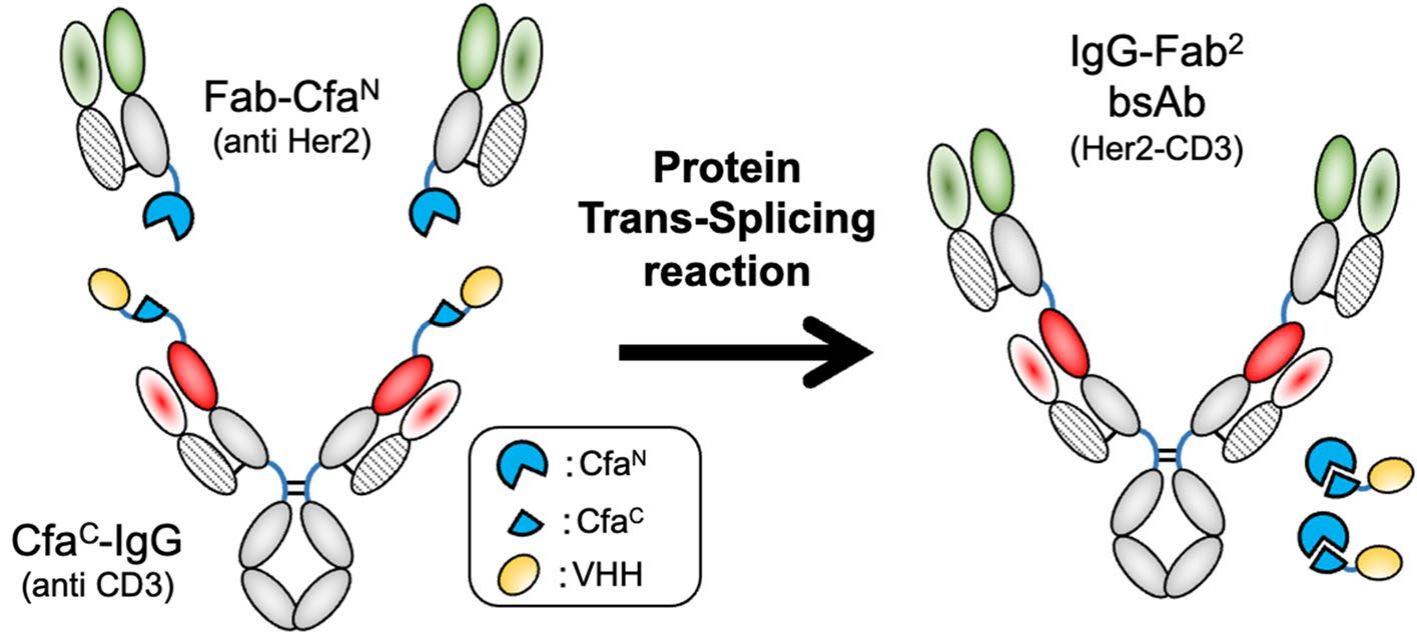
Reaction scheme of IgG–Fab^2^ construction via PTS reaction.

This study utilized the PTS reaction to construct the IgG–Fab^2^ bsAb (Fig. 1). The IgG–Fab^2^ format was initially developed to construct multivalent mono-specific antibodies^16^. The heavy chain-light chain-pairing problem, caused by the similarity of two different light chains, humpers its construction by the general recombinant expression method although IgG–Fab^2^ is an interesting format for bsAb. Thus, the use of a common light chain^17^ or exchanging one light chain with one of the VH-CH1 portions, FIT-Ig, was previously reported to overcome the mispairing issue^18,19^. Obtaining the common light chain is a cumbersome process and the FIT-Ig production potentially results in undesired Fab formation although these techniques are interesting. In this study, we report the PTS-based method for the IgG–Fab^2^ bsAb production by ligating the separately prepared IgG portion and the Fab portion. The heavy chain/light chain-pairing problem was avoided because the IgG part and Fab parts were separately expressed. Here, we demonstrate the construction of IgG–Fab^2^ bsAb, which binds to the Her2 and CD3 antigens.

## Results and discussion

### Design of IgG and Fab parts for IgG–Fab^2^ bsAb

We designed intein-C fused to the N-terminus of the heavy chain of an IgG and intein-N fused to the C-terminus of the CH1 domain of a Fab to construct the IgG–Fab^2^ bsAb molecule by the split-intein–mediated ligation (Fig. 1). This process can separately express, purify, and connect these two portions by the split intein–mediated PTS reaction (Fig. 1). We chose Cfa DnaE split intein for this purpose, which is widely used for several protein engineering applications, including antibody labeling^20^ and bsAb constructions^21^. An anti-CD3 (M291) IgG^22^ and an anti-Her2 (trastuzumab)^23^ Fab were selected to construct IgG–Fab^2^. The anti-Her2/anti-CD3 bsAb is one of the popular constructs as a therapeutics for breast cancer treatment^24–26^. A VHH (Ia1; an anti-EGFR VHH)^27^ was fused to the N-terminus of intein-C of Cfa intein (Cfa^C^) to improve the expression. The intein-N of Cfa intein (Cfa^N^) was fused to the C-terminus of the CH1 domain of the trastuzumab Fab.

### The PTS reaction for IgG–Fab^2^ (Her2/CD3)

**Figure 2:**
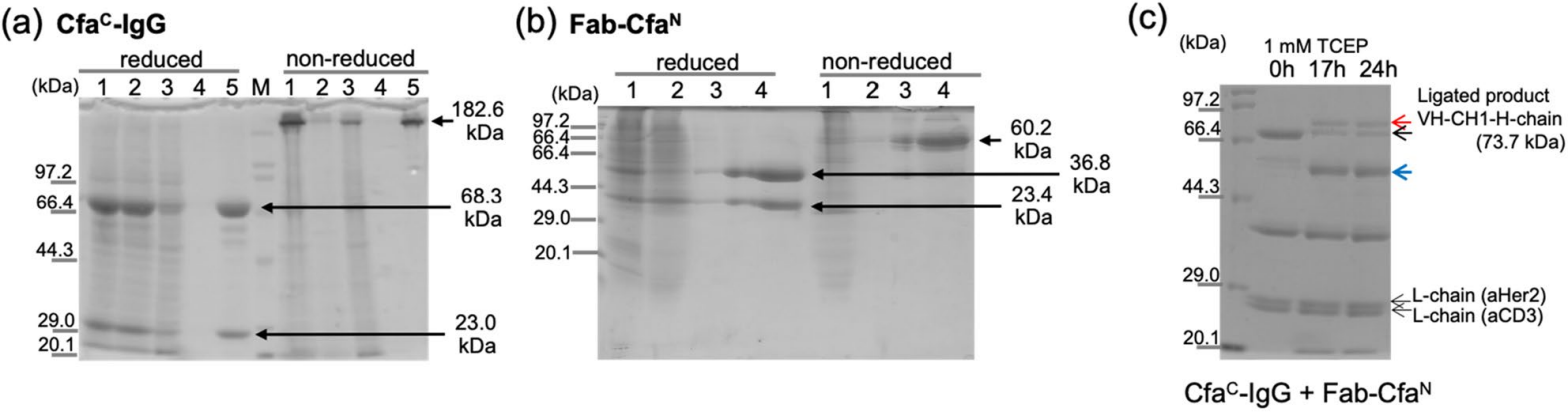
Expression, purification, and ligation of Cfa^C^-IgG and Fab-Cfa^N^. (**a**), and (**b**) reduced and non-reduced SDS-PAGE for purification of Cfa^C^-IgG and Fab-Cfa^N^ using a protein A column. (**a**) 1: culture medium, 2: pre-column, 3: flowthrough, 4: wash, 5: elute. M: maker. (**b**) 1: pre-column, 2: flowthrough, 3: wash, 4: elute. M: molecular-weight maker. (**c**) SDS-PAGE results for the PTS reactions between Cfa^C^-IgG and Fab-Cfa^N^. A top black arrow indicates unreacted Cfa^C^-heavy chain. Red arrow indicates bands for the ligated product (VH-CH1-H chain). A blue arrow indicates heavy chain generated by the uncontrolled cleavage at the C-terminal side of Cfa^C^.

**Figure 3:**
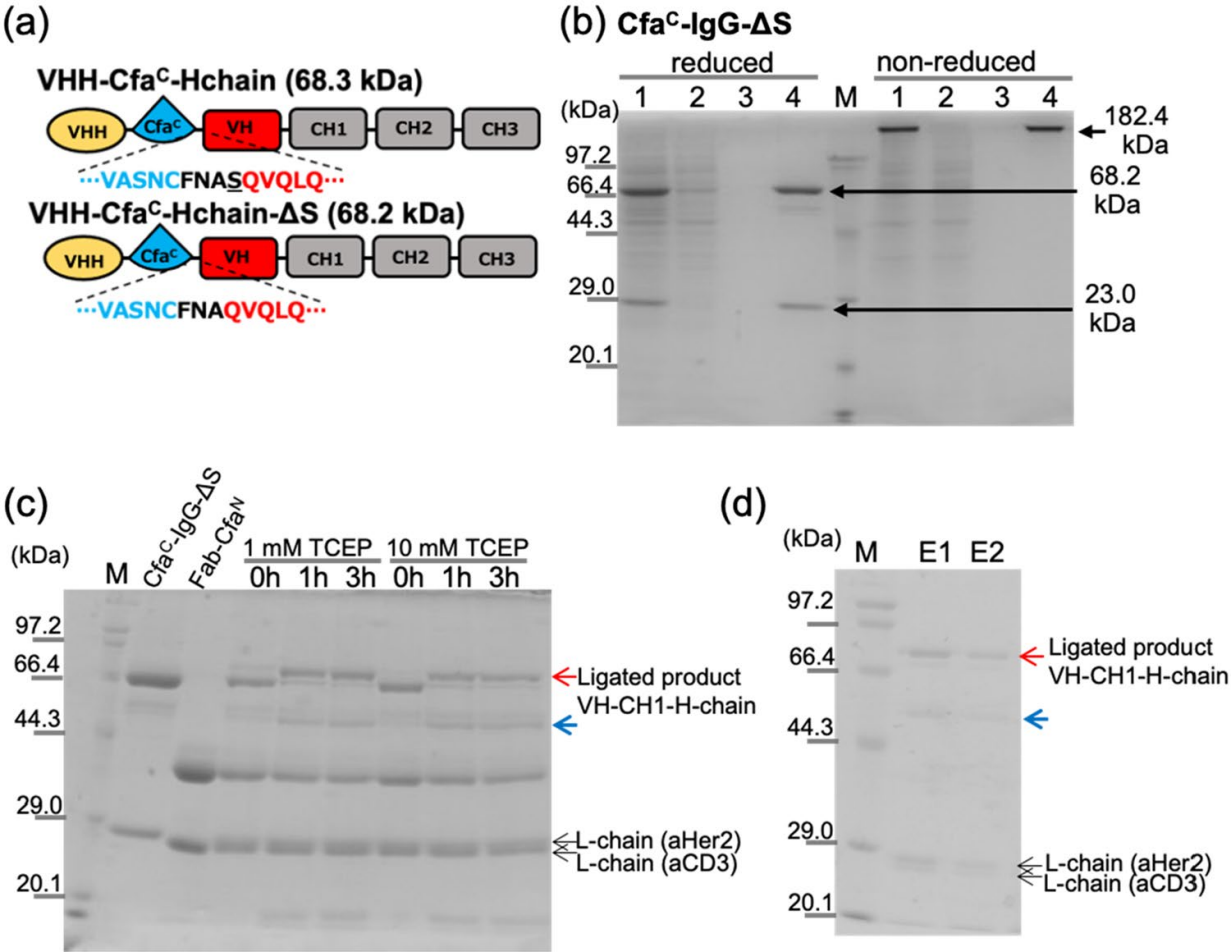
Expression, purification, and ligation of Cfa^C^-IgG-ΔS. (**a**) Domain order of heavy chain of VHH-Cfa^C^-Hchain and VHH-Cfa^C^-Hchain-ΔS. Amino acid sequence between Cfa^C^ and VH are shown. (**b**) Reduced and non-reduced SDS-PAGE for purification of Cfa^C^-IgG-ΔS using Ni–NTA column. 1: pre-column, 2: flowthrough, 3: wash, 4: elute. M: molecular-weight maker. (**c**) SDS-PAGE result for the PTS reactions between Cfa^C^-IgG-ΔS and Fab-Cfa^N^. (**d**) SDS-PAGE result of the elution fraction (E1 and E2) of anion exchange column. The reaction mixture of the PTS reaction between Cfa^C^-IgG-ΔS and Fab-Cfa^N^ was applied to the column. E1 and E2: Elution fractions, M: molecular-weight maker. Red arrows indicate bands for the ligated product (VH-CH1-H-chain). Blue arrows indicate heavy chain generated by the uncontrolled cleavage at the C-terminal side of Cfa^C^.

Cfa^C^-IgG (anti-CD3) and Fab-Cfa^N^ (anti-Her2) were expressed by a mammalian cell expression system and purified by protein A and Ni–NTA columns for Cfa^C^-IgG and Fab-Cfa^N^, respectively. Figure 2a, b show an SDS–polyacrylamide gel-electrophoresis (SDS-PAGE) image of protein purification. Non-reduced SDS-PAGE showed that interchain disulfide bonds were formed for both molecules shown by single bands at elute lanes (182.6 kDa for Cfa^C^-IgG and 60.2 kDa for Fab-Cfa^N^). The purified proteins were then mixed to perform the PTS reaction with a 1:2 molar ratio of Cfa^C^-IgG and Fab-Cfa^N^ because there are two reaction sites and thus the reaction point for the PTS reaction is equimolar by mixing with a 1:2 molar ratio. A new band at high molecular weight appears after 17 h that corresponds to the ligated product of VH-CH1 of anti-Her2 and heavy chain of anti-CD3 (73.7 kDa; red arrow in Fig. 2c). Approximately 50% of the band of VHH-Cfa^C^-Hchain (black arrow in Fig. 3a) remains after 24 h reaction although the ligation reaction proceeded. A band corresponds to the heavy chain cleaved at the C-terminal side of Cfa^C^ because the side reaction of the PTS reaction was observed in addition to the imperfect reaction. Such an undesired side reaction was commonly observed in the split intein-mediated PTS reaction. We examined the sequence around the intein reaction site to identify the causative factor for the low reaction yield and side cleavage reaction, and we revealed a possible glycosylation site at the extein portion at the C-terminal side of Cfa^C^ (Fig. 3a). The NheI cloning site introduced a “ ~ AS ~ ” sequence directly before the VH gene (anti-CD3), and the N-linked glycosylation consensus sequence formed, Asn-X-Ser/Thr^28^. Thus, we speculated that the possible N-linked glycosylation at the Ser portion caused the low reaction yield and side cleavage reaction.

Therefore, we constructed an expression vector for the Ser deletion mutant at the glycosylation site, termed VHH-Cfa^C^-Hchain-ΔS (Fig. 3a). We expressed and purified Cfa^C^-IgG-ΔS (anti-CD3) similarly with Cfa^C^-IgG (Fig. 3b). SDS-PAGE showed that the Cfa^C^-IgG-ΔS was purified after the protein A column purification with a single band in the non-reduced condition (Fig. 3b; 182.4 kDa).

Then, we performed the PTS reaction between Cfa^C^-IgG-ΔS and Fab-Cfa^N^ (Fig. 3c). The concentration effect of reducing reagent TCEP (Tris(2-carboxyethyl)phosphine)) during the PTS reaction was evaluated by performing 1 mM and 10 mM TCEP conditions (Fig. 3c). Both TCEP conditions demonstrated no significant difference of the reaction yield. The use of the serine deletion mutant (Cfa^C^-IgG-ΔS) showed the reduction of the side-reaction product of the uncontrolled cleavage and the improvement of the ligation yield compared with the original Cfa^C^-IgG and Fab-Cfa^N^ reaction. Although we set the reaction stoichiometry 1:2 for Cfa^C^-IgG-ΔS and Fab-Cfa^N^, the band for the Fab-Cfa^N^ remained after the ligation reaction while the band for Cfa^C^-H-chain mostly converted to the ligated product, VH-CH1-H-chain (Fig. 3c). We speculated that the reason for the residual reactant of Fab-CfaN after the ligation reaction could be a result of the amount excess of the Fab-CfaN compared with CfaC-IgG-ΔS because of the inaccuracy of concentration determination using UV. The reaction mixture was applied to the Ni–NTA column to remove the unreacted Fab-Cfa^N^ then the flowthrough fraction was applied to an anion-exchange chromatography column. The SDS-PAGE showed the purified target bsAb molecule with a trace amount of the cleaved side product (Fig. 3d). Hereafter, we termed the target bsAb molecule with the ΔS mutation as IgG–Fab^2^(Her2/CD3).

### Binding activity of IgG–Fab^2^(Her2/CD3)

**Figure 4:**
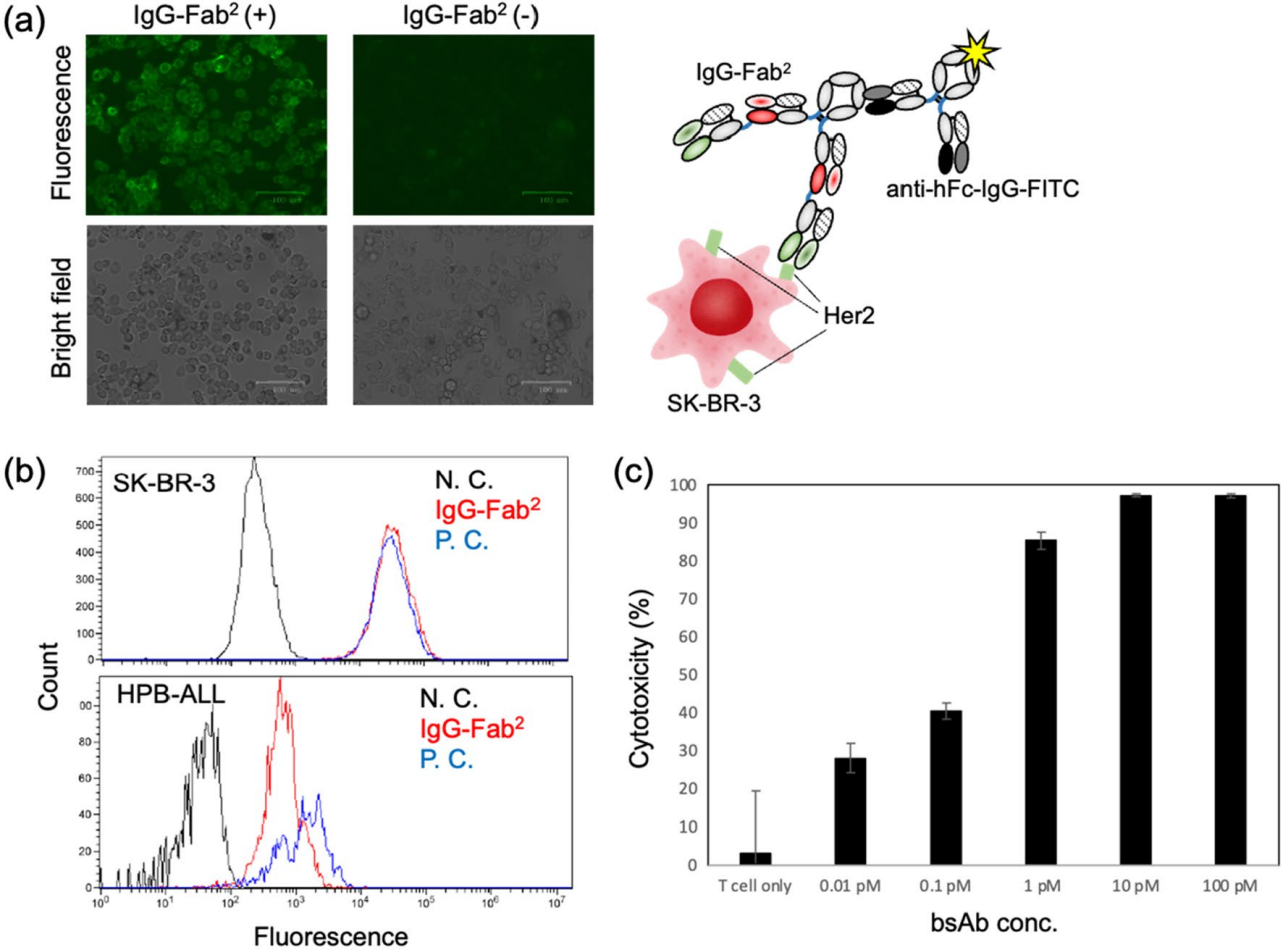
(**a**) Fluorescent microscopy image of SK-BR-3 cells. Top panels: fluorescent images. Bottom panel: bright field images of the same observation areas of the fluorescent images. The pictures indicate 100-μm scale bars. Antibody combinations for fluorescence imaging is illustrated on the right. (**b**) Flowcytometry analyses using Her2 positive (SK-BR-3) or CD3 positive (HPB-ALL) cells. Black: negative control (w/o antibody). Blue: positive control using anti-Her2 or anti-CD3 antibodies. Red: IgG–Fab^2^. (**c**) In vitro cytotoxicity assay. Target cell: Her2^+^ SK-BR-3 cell. Effector cell: LAK T-cell. E/T ratio = 5. Standard deviations of the determined cytotoxicity values are indicated with error bars (n = 4).

The binding activity of the constructed IgG–Fab^2^(Her2/CD3) was evaluated. First, we observed the IgG–Fab^2^ binding to Her2-positive cells by fluorescent microscopy (Fig. 4a). A clear fluorescence was obtained on the cell surface for the sample treated with bsAb compared with the fluorescent microscopy image without adding bsAb. The fluorescence is emitted from a FITC-labeled anti-Fc antibody, and the anti-Her2 Fab portion locates the N-terminal side of IgG–Fab^2^. Thus, the fluorescent image indicates not only the binding activity to the Her2 antigen but also the correct structure formation of the Fab (Her2)-Fab (CD3)-Fc structure (Figs. 1, 4a). Then, we evaluated the binding activity by flow cytometry using Her2-positive cells and CD3-positive cells, and the IgG–Fab^2^(Her2/CD3) bsAb demonstrated a binding activity toward both cells (Fig. 4b). These results revealed the binding activities of IgG–Fab^2^(Her2/CD3) toward Her2 and CD3.

### Cytotoxic activity of IgG–Fab^2^(Her2/CD3) mediated by activated T cells

An in vitro cytotoxicity assay measured the cancer cell-killing activity of IgG–Fab^2^(Her2/CD3) mediated by activated T cells (lymphokine-activated killer cells with the T-cell phenotype [T-LAK]). The Her2-positive SK-BR-3 cells were incubated with activated T cells containing different IgG–Fab^2^(Her2/CD3) concentrations (Fig. 4c). The effector cells/target cells (E/T) ratio was set to 5. Over 80% cell killing was observed at a 1 pM concentration of bsAb. This indicates that the constructed IgG–Fab^2^(Her2/CD3) molecule simultaneously bridges an SK-BR-3 cell and an activated T cell through the binding of the Her2 and CD3 antigens. It is known that the Her2/CD3 antibody induced T-cell activation through cytokine production, and we expected a similar mechanism should be induced by IgG–Fab^2^(Her2/CD3) in our cytotoxicity experiment.

## Conclusions

An advantage of the IgG–Fab^2^ bsAb over the general IgG type bsAb is the bivalency for the two targets. Because of the bivalency, the IgG–Fab^2^ bsAb can bind to each target strongly through the avidity effect, which may contribute to better therapeutic outcomes. Here, we demonstrated the novel bsAb construction method with the format of IgG–Fab^2^ using the PTS reaction to achieve a specific pairing between the heavy chain and the light chain of two Fab parts. Previous studies used the common light chain^17^ or connecting light chain to the N-terminus of IgG heavy chain, FIT-Ig^18,19^, to construct IgG–Fab^2^ type bsAb. Obtaining the common light chain is a cumbersome process and FIT-Ig production potentially generates mispaired byproduct Fab. Our method can prevent such production problems by separating the IgG part and the Fab part productions. Additionally, our method can be utilized for screening tasks to evaluate several IgG and Fab part combinations. Any combinations can be generated by mixing Cfa^C^-IgG and Fab-Cfa^N^, once several clones of Cfa^C^-IgG and Fab-Cfa^N^ are constructed. The use of the split intein–based protein ligation method for bsAb production will expand our bsAb construction strategy, and highly efficient cancer-treating drugs will be obtained based on this technology.

## Materials and methods

### Expression and purification of proteins

The Expi293 mammalian cell expression system (Thermo Fisher Scientific, MA USA) with HE400 medium (GMEP, Fukuoka, Japan) was used to produce recombinant proteins. Expression vectors of VHH-Cfa^C^ fused heavy chain gene and light chain gene of M291 antibody were simultaneously transfected to the cell using PEI MAX reagent (Polyscience Inc., PA USA) for Cfa^C^-IgG expression. Expression vectors of Cfa^N^ fused VH-CH1 gene and the light chain gene of trastuzumab for Fab-Cfa^N^ were transfected similarly for Cfa^C^-IgG expression. The supernatants were collected and applied to the purification columns 7 days after culture. Protein A column (UNOsphere SUPrA, Biorad, CA USA) was used for the Cfa^C^-IgG purification and Ni–NTA column (FUJIFILM Wako chemicals, Osaka, Japan) for the Fab-Cfa^N^ purification.

### PTS reaction

Purified Cfa^C^-IgG and Fab-Cfa^N^ samples were mixed in a PTS ligation buffer (50 mM HEPES, pH: 7.0, 200 mM of NaCl, 1 or 10 mM TCEP) at 37 °C. The final concentrations of Cfa^C^-IgG and Fab-Cfa^N^ were 2 μM and 4 μM, respectively. The reaction mixture was subjected to the Ni–NTA column and the flowthrough fraction was collected after 3 h of the ligation reaction. AKTA start chromatography system with a 5 mL of HiPrep Q HP column (Cytiva, MA USA) was used for anion-exchange chromatography.

### Flow cytometry

For 15 min on ice, 10^6^ cells of SK-BR-3 (Her2^+^) (obtained from ATCC) or HPB-ALL (CD3^+^) (obtained from Cell resource center for biomedical research, Tohoku university; ID: TKG 0199) were incubated with 30 nM of IgG–Fab^2^ in phosphate-buffered saline (PBS). An anti-human Fc FITC conjugate antibody (Sigma-Aldrich, MO USA) was added after washing the cells with PBS twice. PBS-suspended cells were used for the analysis using an RF-500 flow cytometry machine (Sysmex, Kobe, Japan) after washing the cells with PBS twice. The analyses used 100 μL of cell suspensions for each measurement. FCSalyzer software (Souceforge; https://sourceforge.net/projects/fcsalyzer/) was used to analyze the obtained data. As positive controls, trastuzumab (Chugai Pharmaceutical Co., Ltd, Tokyo, Japan) and anti-CD3 FITC (Proteintech Group, Inc., IL, USA) were used for SK-BR3 and HPB-ALL, respectively.

### Fluorescent microscopy

SK-BR-3 cells were cultured on a 24-well cell culture dish in Dulbecco’s Modified Eagle Medium medium with 10% fetal bovine serum. IgG–Fab^2^ at 30 nM in PBS was added and incubated for 20 min after removing the medium. Then, an anti-human Fc FITC conjugate antibody (Sigma-Aldrich, MO USA) was added after washing the wells with PBS twice. Cells were observed by ZOE fluorescent imager (Biorad, CA USA) after washing the wells. No antibody was added for the control cells.

### In vitro cytotoxicity assay

T-LAK cells were induced as described in the literature^29^. Peripheral blood mononuclear cells (CTL-UP1 uncharacterized PBMC) were cultured for 48 h at a density of 1 × 10^6^ cells/mL in a medium supplemented with 100 IU/mL of recombinant human interleukin 2 (Shionogi Pharmaceutical Co.) in a culture flask (A/S Nunc) pre-coated with anti-CD3 monoclonal antibody (10 μg/mL). The in vitro growth inhibition of cancer cells using Her2 positive SK-BR-3 cells was assayed using an MTS assay kit (CellTiter 96® AQueous Non-Radioactive Cell Proliferation Assay; Promega) with the E/T ratio set to 5, as reported in the literature^29^.

### Supplementary Information

Supplementary Information.

## Data Availability

All raw image data used for SDS-PAGE and fluorescent microscopy are included in the supplementary information file. The other datasets used and/or analysed during the current study available from the corresponding author on reasonable request.
