# Supplementary material for: Construction of IgG–Fab2 bispecific antibody via intein-mediated protein trans-splicing reaction
Source: Sci Rep. 2023 Sep 25;13:15961. doi: 10.1038/s41598-023-43110-0 (PMC10520027; doi:10.1038/s41598-023-43110-0)

Supplementary information

Raw SDS-PAGE gel images for Figure 2

(a)

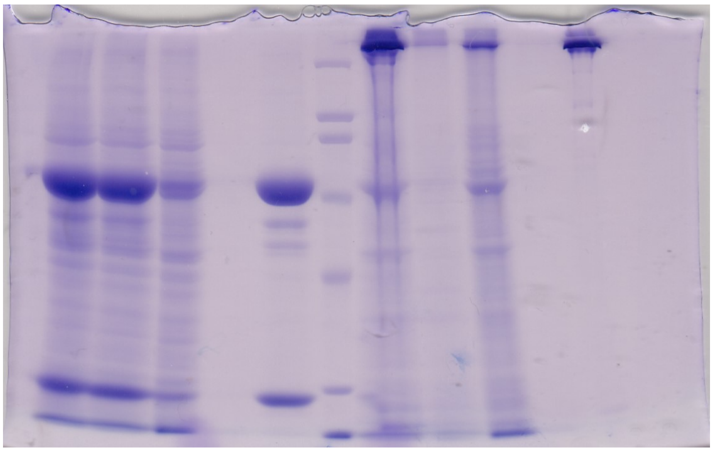

(b)

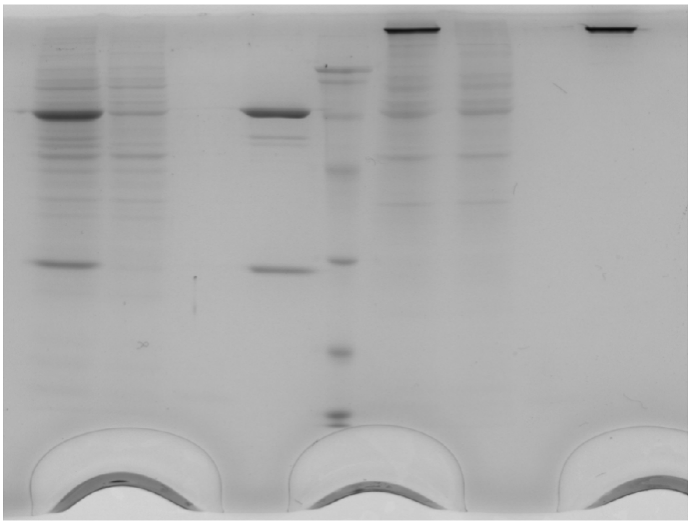

(c)

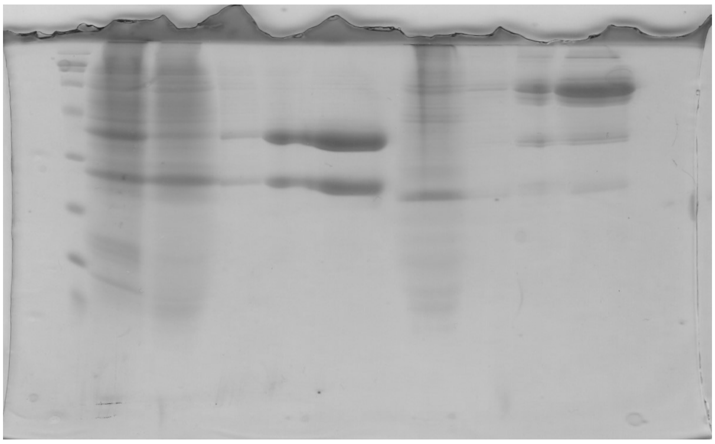

Raw SDS-PAGE gel images for Figure 3

(a)

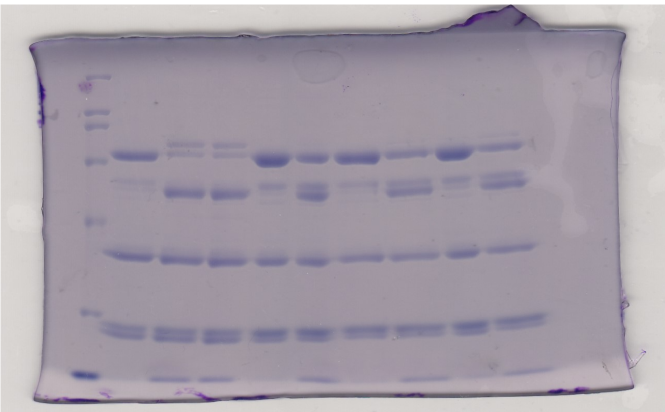

(b)

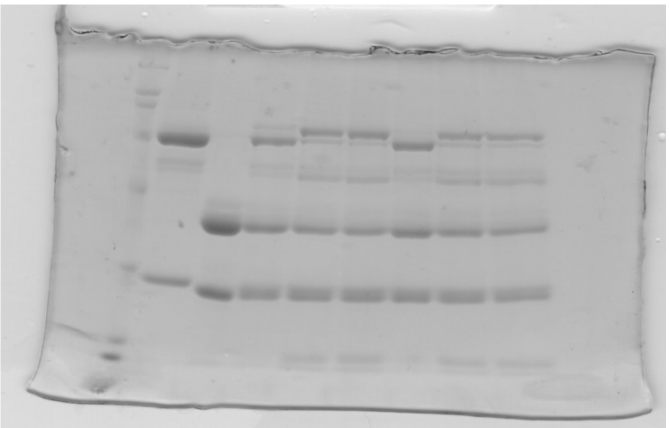

(c)

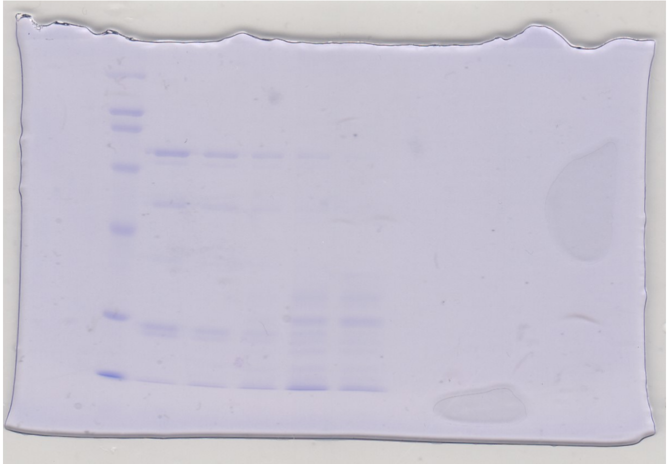

Raw fluorescent microscopy images for Figure 4(a)

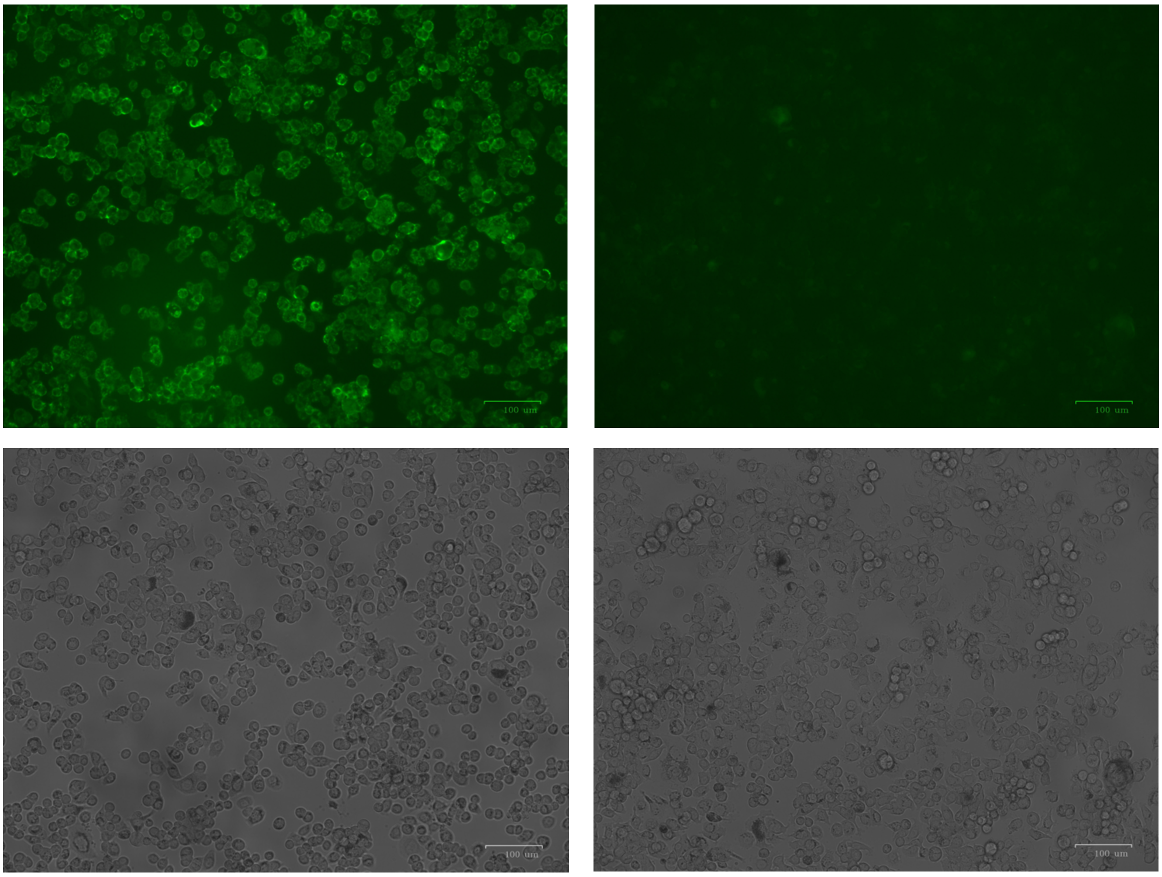

Supplement: Supplementary file 1 — Supplementary Information. [file 41598_2023_43110_MOESM1_ESM.pdf]
